# Large gastroduodenal artery pseudoaneurysm and arterioportal fistula in chronic pancreatitis

**DOI:** 10.1016/j.radcr.2024.08.038

**Published:** 2024-09-07

**Authors:** Caroline J. Cushman, Andrew F. Ibrahim, Thomas Callahan

**Affiliations:** a School of Medicine, Texas Tech University Health Sciences Center, Lubbock, TX, USA; b Department of Interventional Radiology, Texas Tech University Health Sciences Center, Lubbock, TX, USA

**Keywords:** Gastroduodenal artery, Pseudoaneurysm, Arterioportal fistula, Pancreatitis

## Abstract

Visceral artery pseudoaneurysms, particularly those in the gastroduodenal artery (GDA), are rare but serious complications associated with chronic pancreatitis, posing a significant risk of rupture due to their structural fragility. In this case, a 61-year-old male with a history of chronic pancreatitis, alcohol cirrhosis, duodenal ulcer, and COPD presented with persistent abdominal pain and recurrent fevers. Imaging revealed a 7 cm pseudoaneurysm between the GDA and superior mesenteric vein, which was successfully treated with coil embolization. This case highlights the importance of prompt recognition and intervention in managing GDA pseudoaneurysms, particularly when complicated by an arterioportal fistula, and demonstrates the efficacy of endovascular therapy as a minimally invasive treatment option that can significantly improve patient outcomes in complex vascular complications associated with chronic pancreatitis.

## Introduction

Visceral artery aneurysm, including true aneurysm and pseudoaneurysm, is a rare disease, with an incidence of 0.01%-0.2% [1]. Visceral artery pseudoaneurysms coupled with chronic pancreatitis, have an increased incidence varying from 1.3% to 10% across different case series [2]. Among visceral artery pseudoaneurysms, the splenic artery is the most frequently involved (60%), followed by the hepatic artery (20%) and the superior mesenteric artery (5.5%) with the gastroduodenal artery accounting for only 1.5% of cases [3]. A pseudoaneurysm is an abnormal outpouching or dilation of an artery, bounded only by the tunica adventitia. They typically occur when there is a breach in the vessel wall, allowing blood to leak through the inner wall but remain contained by the adventitia or perivascular soft tissue. This can result from inflammation, vasculitis, iatrogenic trauma, or infection [4]. Unlike true aneurysms, which involve the dilation of all 3 layers of the vessel wall and are often caused by atherosclerosis and hypertension, pseudoaneurysms are caused by abdominal trauma (such as pancreatic surgery) or inflammatory sequelae (like pancreatitis) and consequently have a higher risk of rupture [5], [6], [7], [8]. The diagnosis of visceral artery pseudoaneurysms is made by CT of upper abdomen with contrast and ultrasonography (USS) and confirmed by angiography [9]. The management of pseudoaneurysms depends on the hemodynamic stability of the patient, their overall status, the anatomy, and the expertise of the treating center [10]. Endovascular therapy with coil embolization, with or without stent placement, is considered the first-line treatment due to its reduced morbidity and mortality compared to open surgery [11], [12], [13]. Thrombin injection can be used less commonly [14,15]. In cases where endovascular treatment fails or the patient is hemodynamically unstable with a perforated aneurysm, a surgical approach is preferred [16].

Arterioportal fistula (APF) is a rare vascular disorder of the abdominal viscera, characterized by arteriovenous communications between the splanchnic arteries and the portal vein or its tributaries [17]. They can be either acquired or congenital and can present with a myriad of clinical manifestations due to their unparalleled structural characteristics and varying pressure dynamics [18]. Visceral artery pseudoaneurysm and APF occur together in rare cases.

## Case presentation

**Fig. 1:**
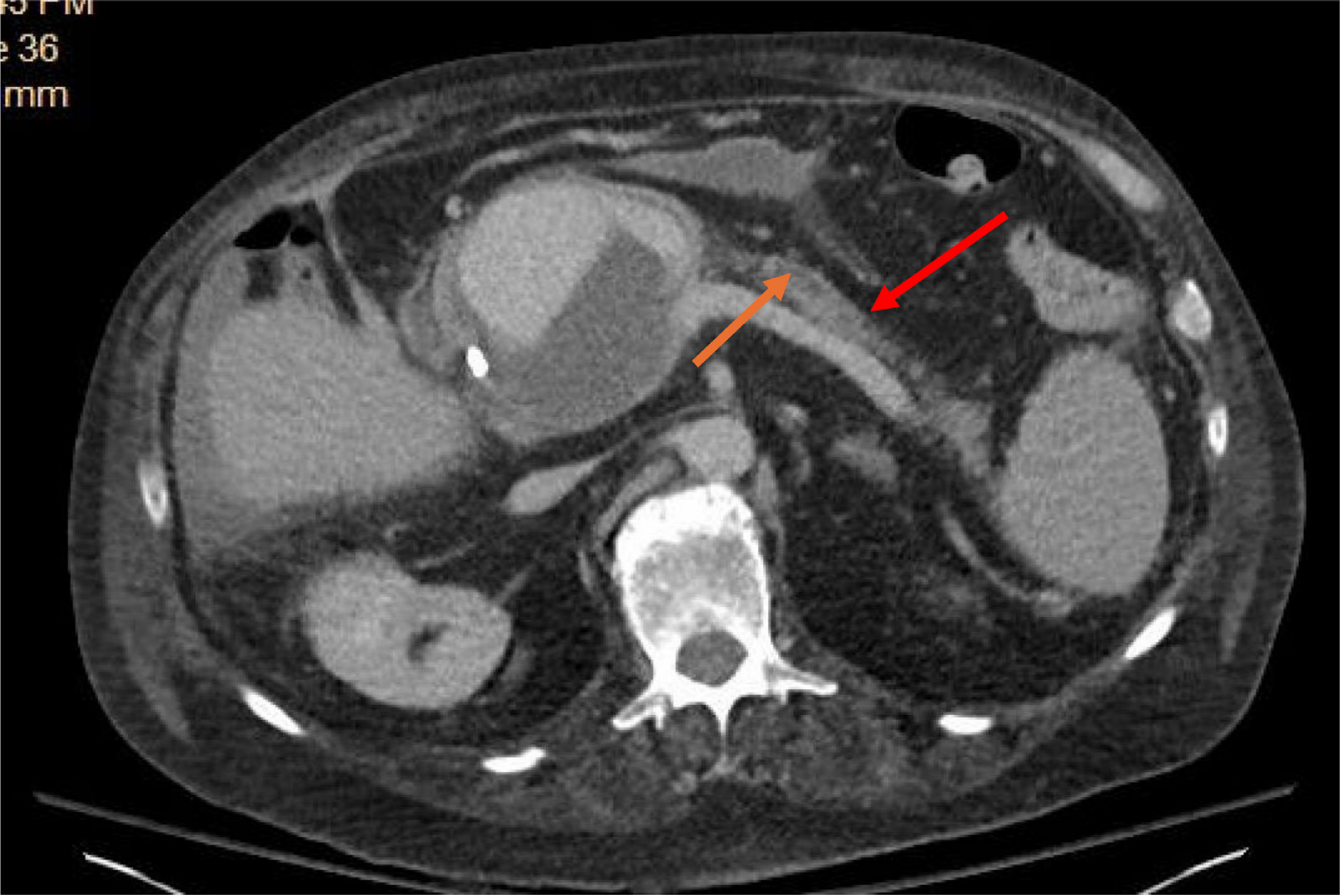
This figure illustrates the profound manifestations of chronic pancreatitis. The red arrow demarcates the remnants of the pancreas, emphasizing the extensive atrophy and damage caused by advanced pancreatitis, while the orange arrow highlights the pancreatic duct.

**Fig. 2:**
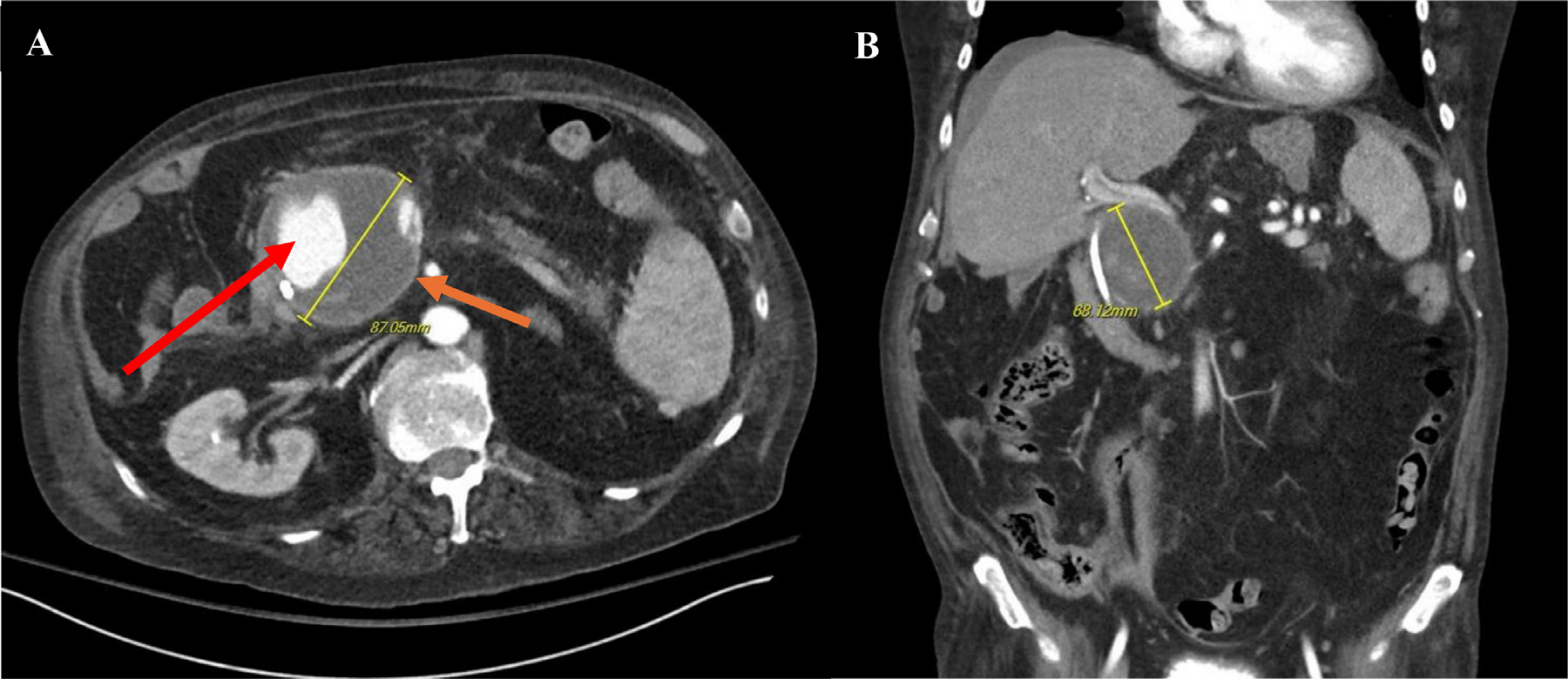
(A and B**)** The orange arrow highlights a large gastroduodenal pseudoaneurysm, as visualized in both axial and coronal CT images. In the axial plane, the pseudoaneurysm measured 87.05 mm, while in the coronal plane, it measured 68.12 mm. The red arrow indicates the presence of radiopaque contrast material within the pseudoaneurysm cavity and the surrounding hypodense region represents chronic thrombosis.

A 61-year-old male with a history of chronic pancreatitis, alcohol cirrhosis, duodenal ulcer, and chronic obstructive pulmonary disease (COPD), presented to the emergency department for persistent abdominal pain and recurrent fevers (Fig. 1). Examination revealed the acute onset of epigastric pain radiating to the back, worsening anemia, and the development of a pseudoaneurysm between the gastroduodenal artery and the superior mesenteric vein (Figs. 2A and B). On admission, the patient's hemoglobin levels were significantly reduced, necessitating multiple transfusions of packed red blood cells. The patient was also started on IV proton pump inhibitors for gastrointestinal protection.

**Fig. 3:**
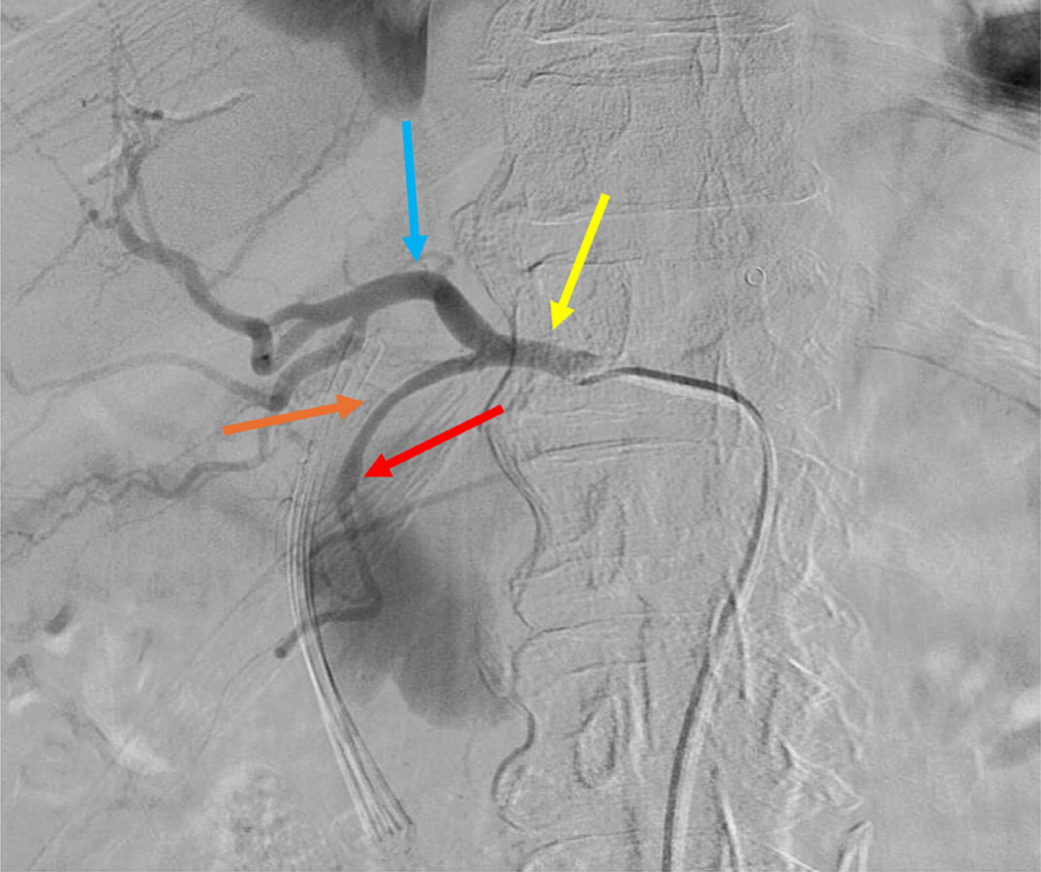
Angiographic image highlighting key vascular structures. The yellow arrow indicates the common hepatic artery, the blue arrow points to the proper hepatic artery, and the orange arrow identifies the gastroduodenal artery (GDA). The red arrow marks the neck of the GDA pseudoaneurysm. An arterioportal fistula (APF) is noted between the gastroduodenal artery and the superior mesenteric vein (SMV).

**Fig. 4:**
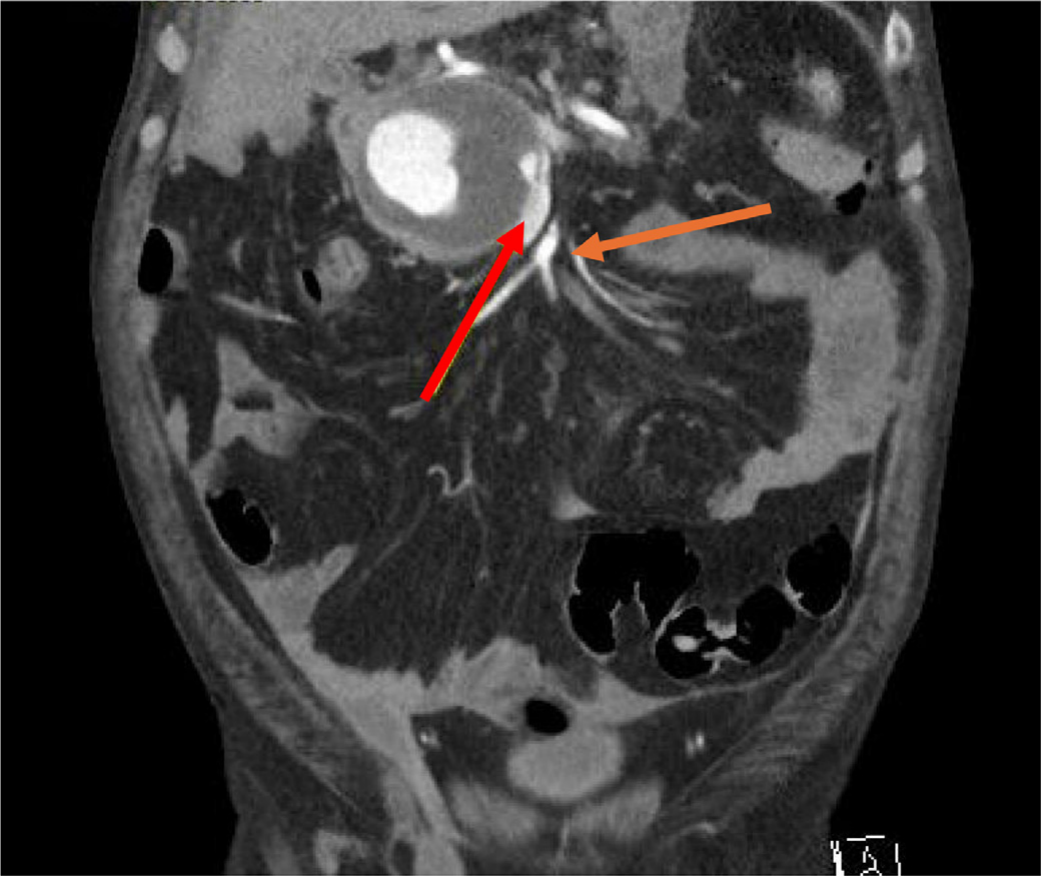
The red arrow indicates the superior mesenteric vein, while the orange arrow marks the superior mesenteric artery. An arterioportal fistula (APF) is observed between the gastroduodenal artery and the superior mesenteric vein (SMV).

Initial imaging, which included a contrast-enhanced CT scan of the abdomen, revealed a 7 cm pseudoaneurysm originating from the gastroduodenal artery and forming a fistula to the superior mesenteric vein (Fig. 3, Fig. 4). There was also a small pseudocyst within the area of the pancreatic head which was insignificant to the case. Given the risk of rupture and the complexity of the vascular involvement, interventional radiology was consulted for embolization of the pseudoaneurysm.

**Fig. 5:**
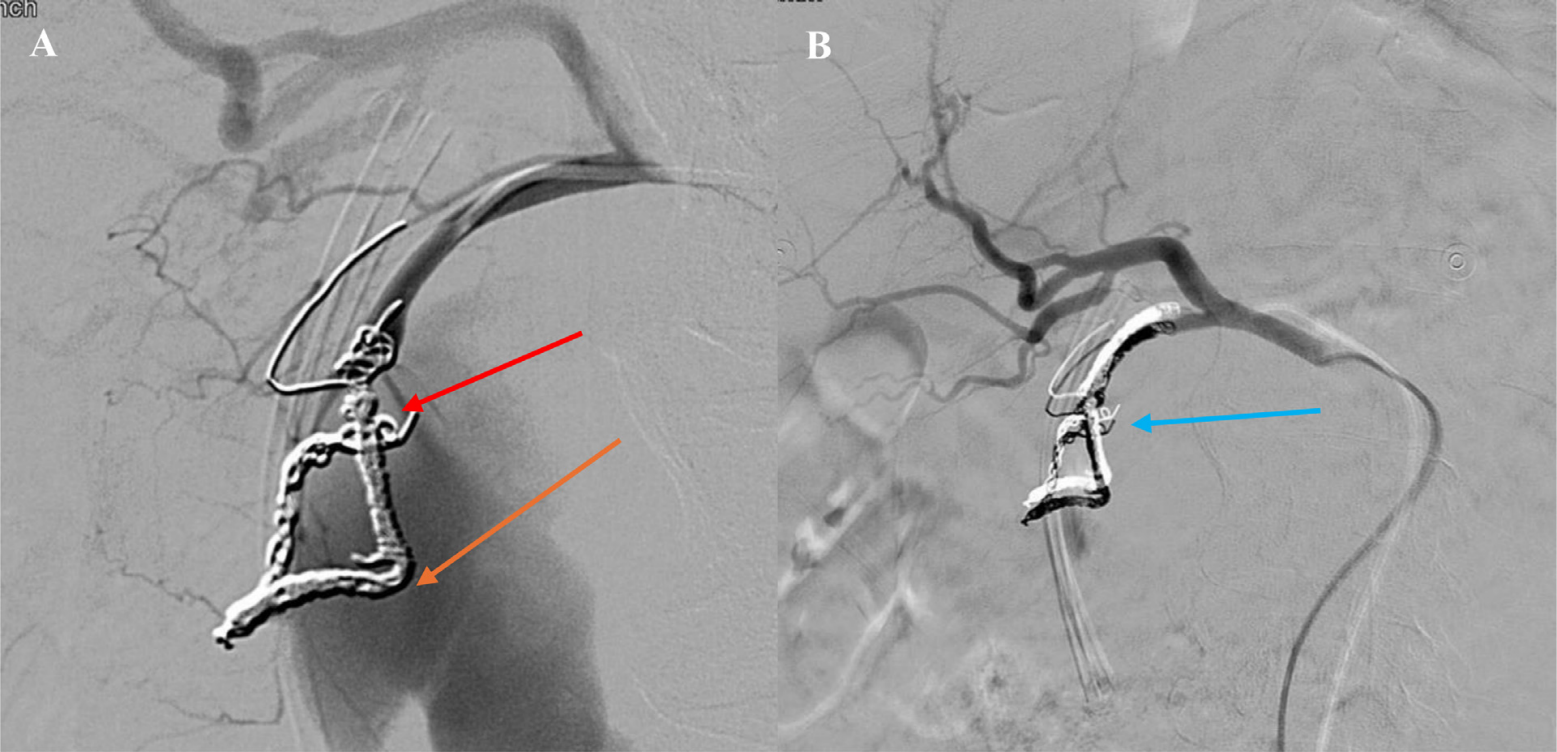
(A and B) Intraoperative fluoroscopic angiogram illustrating coil placements during transarterial embolization (TAE). The orange arrow indicates coils placed in the gastroepiploic artery, the red arrow points to packing coils at the neck of the pseudoaneurysm, and the blue arrow identifies coils within the gastroduodenal artery (GDA). Ruby and packing coils were deployed to occlude the arterioportal fistula and achieve complete embolization.

The embolization was performed the day following the patient's admission. Under ultrasound guidance, the right common femoral artery was accessed, and a 5 French micropuncture kit was used to place a catheter into the aorta. The celiac artery was selectively catheterized using a Cobra glide catheter and glide wire. Multiple arteriograms confirmed the presence of a large pseudoaneurysm arising from the gastroduodenal artery with a well-defined neck. A 5 French catheter was advanced past the neck of the aneurysm into the gastroepiploic artery, and embolization was performed using multiple coils. A 5 × 15 mm Ruby coil and a 4 × 10 mm packing coil were placed distal to the aneurysm neck, ensuring complete occlusion (Figs. 5A and B). Postembolization arteriograms showed no further arterial flow to the pseudoaneurysm, confirming the success of the procedure.

**Fig. 6:**
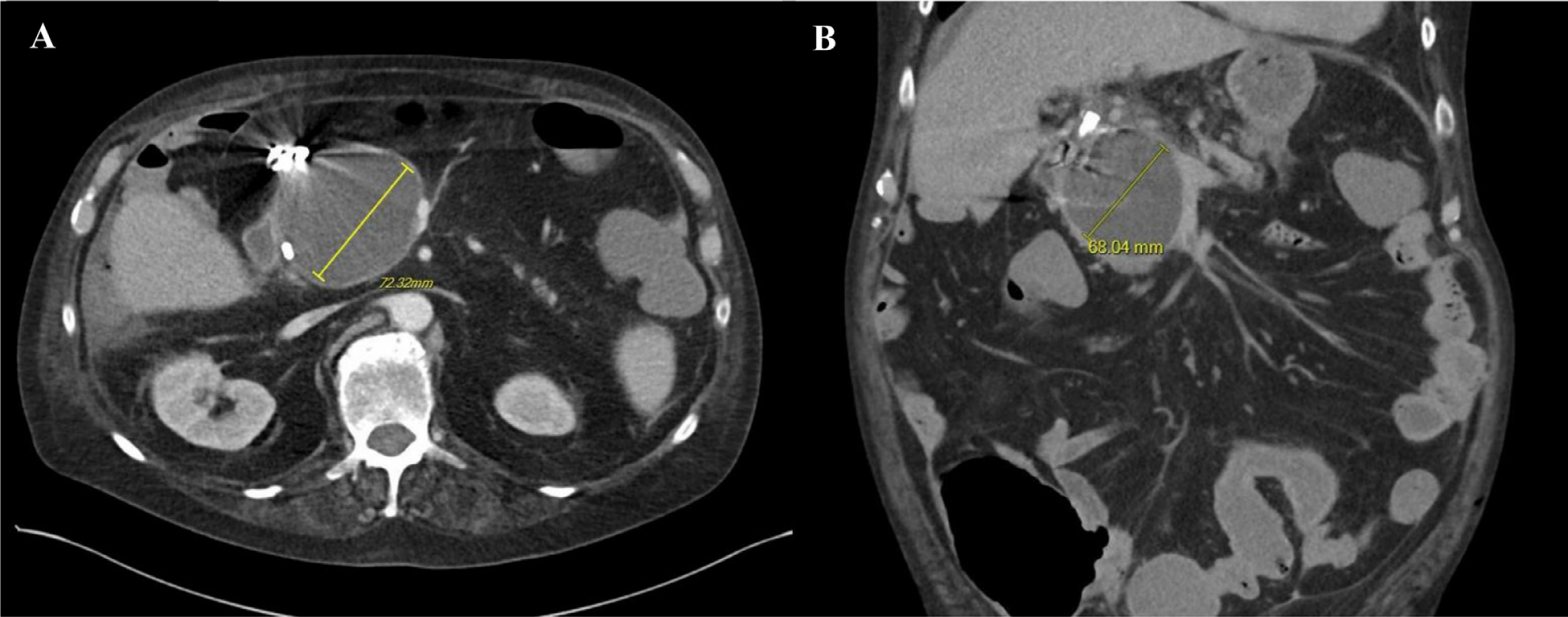
(A and B) Postoperative pseudoaneurysm visualized on axial and coronal CT images. A solid hypodense area corresponds to the neck of the gastroduodenal artery pseudoaneurysm, which has been successfully occluded with embolization packing and Ruby coils. No residual arterioportal fistula is evident. The pseudoaneurysm measured 72.32 mm in the axial plane and 68.04 mm in the coronal plane, indicating regression from embolization interventions performed.

The patient tolerated the procedure well, with subsequent improvement in hemoglobin levels and resolution of abdominal pain. Follow-up CT imaging confirmed complete thrombosis of the pseudoaneurysm with no active contrast extravasation (Figs. 6A and B). The patient was closely monitored and continued on supportive care, including IV albumin and diuretics for acute kidney injury and hypotension. His cardiac and pulmonary status were stabilized with medical management, and his gastrointestinal symptoms showed marked improvement. The patient was discharged in stable condition. He was hemodynamically stable, and able to eat and drink without nausea, vomiting, or pain.

## Discussion

In the current literature, there is one analogous article describing similar disease pathology in which a gastroduodenal artery (GDA) pseudoaneurysm with arterioportal fistula (APF) coexisted in a patient with chronic pancreatitis. However, this case also involved portal vein stenosis, which was not present in our case [2]. Another related incident involving chronic pancreatitis described a ruptured pseudocyst that connected the GDA and portal vein [19]. Yet, since this involved a pseudocyst rather than a pseudoaneurysm, it is not directly comparable. The incidence reported in our case is notably exceptional and extremely rare, given the paucity of similar reports in the literature.

Chronic pancreatitis (CP) is a fibro-inflammatory disease characterized by a persistent low-grade inflammatory state [20]. Over time, chronic inflammation leads to parenchymal fibrosis, resulting in the loss of pancreatic endocrine and exocrine function due to irreversible damage to the pancreatic parenchyma and ductal system [21]. In its most severe form, CP can lead to various complications, including pancreatic duct disruption (pseudocyst), obstruction (biliary or duodenal), pancreatic insufficiency (diabetes or exocrine pancreatic insufficiency), and vascular complications (pseudoaneurysms, splenic vein thrombosis, and gastric varices), as well as pancreatic cancer [21]. Visceral artery pseudoaneurysms are uncommon sequelae of both acute and chronic pancreatitis. In pancreatitis, pseudoaneurysms are caused by the erosion of nearby vessels due to the leakage of digestive enzymes [22]. As aforementioned, they are most common in the splenic artery and relatively rare in the gastroduodenal artery (GDA) [2].

An arteriovenous portal fistula (APF) is an abnormal communication between any splanchnic artery or its tributaries and the portal vein, most commonly involving the hepatic artery (65%), followed by the splenic artery (11%) and the superior mesenteric artery (10%) [23]. APFs are more commonly intrahepatic than extrahepatic, noncongenital as compared to congenital and typically secondary to iatrogenic causes such as surgical interventions, including percutaneous or transjugular liver biopsy, TIPS procedures, and transhepatic biliary drainage. They can also occur following nonsurgical incidents such as trauma (including blunt, stab, or gunshot wounds), pseudoaneurysms, and neoplasms such as hepatocellular carcinoma (HCC) or benign tumors. Congenital fistulas, such as those seen in Osler–Weber–Rendu syndrome, are rare, with only 18 cases of congenital intrahepatic AVF reported in the literature [24].

Pseudoaneurysms and AVFs can range from asymptomatic conditions to life-threatening scenarios, such as peritoneal or retroperitoneal hemorrhage [25]. Due to their large shunt flow, AVFs are likely to cause symptoms of presinusoidal portal hypertension [17]. Additionally, portal hypertension occurs in about 15% of patients with chronic pancreatitis due to splenic or portal thrombosis [19].

The diagnosis of complications arising from chronic pancreatitis, such as pseudoaneurysms and arteriovenous fistulas (AVFs), can be achieved using various imaging modalities, including contrast-enhanced CT, magnetic resonance imaging (MRI), ultrasonography with Doppler, and angiography. Contrast-enhanced CT is particularly effective for detecting small aneurysms and assessing anatomical details, as it provides optimal visualization of these vascular abnormalities. In our patient's case, the initial diagnosis was confirmed using contrast-enhanced CT, which revealed acquired extrahepatic AVFs induced by the communication of a gastroduodenal artery (GDA) pseudoaneurysm.

Endovascular treatment has recently become the preferred approach for managing pseudoaneurysms. Surgical intervention should be considered a last resort when interventional radiology is unable to access the pseudoaneurysm. Surgical access can be particularly challenging when the injured vessel lies deep within the pancreatic parenchyma or a pseudocyst, making precise localization difficult [26]. Additionally, dense adhesions and distorted anatomy resulting from long-standing inflammation can further complicate surgical procedures [7]. When deciding between endovascular and surgical therapy, it is essential to consider the vessel anatomy, location, etiology of the disease, and the patient's underlying condition [27].

The elected treatment method for this aneurysm was endovascular transcatheter arterial embolization (TAE) of the neck pseudoaneurysm, gastroduodenal artery, and gastroepiploic artery to prevent backfill. This approach was selected due to its speed, cost-effectiveness, and efficacy, as evidenced by follow-up CT scans showing no blood flow and a contracting pseudoaneurysm.

We employed combined trans-arterial embolization (TAE), targeting the inflow of the pseudoaneurysm and allowing the outflow from the arterioportal fistula (APF) to close on its own. As pressure declines, the outflow tracts clot over, making further intervention unnecessary in our scenario. We used helical ruby and packing coils for embolization, opting for packing coils over n-Butyl cyanoacrylate (NBCA) for occlusion.

In previous literature, a portal vein stent was used to address portal vein stenosis, which inadvertently prevented coil migration during TAE. However, our case differed as there was no stenosis present, and we determined that the packing coils were stable without the need for a stent. A covered stent would be preferred if the targeted vessel was the last supplying the stomach, as coils and glue would completely close the vessel, compromising blood supply. The stomach receives blood supply from 4 main arteries: the left gastric, right gastric, short gastric, and gastroduodenal arteries. In cases of gastric bypass, many of these vessels are severed. Before proceeding with TAE, it is crucial to ensure that the gastroduodenal artery can be sacrificed. As in our case, when the other vessels remain intact and there is adequate collateral blood supply, this approach presents itself as the safest method. Thrombin injection was discussed but deemed unviable due to the arteriovenous fistula connection and the risk of it entering the portal system, potentially causing portal vein thrombosis.

Using only coils within the aneurysm was deemed too costly and ineffective due to its size, as complete coil coverage is generally preferred for smaller aneurysms. Although NBCA was an option, it was not available in-house. Therefore, immediate treatment with TAE was necessary to prevent imminent rupture.

Endovascular treatment has gained traction for addressing various complications of chronic pancreatitis, thanks to numerous advancements in the field over the past decade. Although treatments for pseudoaneurysms and arteriovenous fistulas (APFs) are widely used, our case is uniquely complex due to the simultaneous occurrence of both conditions. Nonetheless, a well-established treatment plan based on the patient's condition and CT imaging can lead to successful outcomes even in complex cases like ours.

## Conclusion

In summary, visceral artery pseudoaneurysms, particularly those associated with chronic pancreatitis, represent a rare but significant clinical challenge with an incidence markedly increased when these conditions coexist. Our case illustrates the complexities involved in diagnosing and managing a gastroduodenal artery pseudoaneurysm complicated by an arterioportal fistula. The utilization of contrast-enhanced CT imaging was crucial for the accurate identification and characterization of these vascular abnormalities. Endovascular trans-arterial embolization (TAE) was successfully employed, demonstrating its efficacy and safety as a first-line treatment modality in such intricate cases. This case underscores the pivotal role of advanced imaging techniques and endovascular interventions in the effective management of pseudoaneurysms and their complications, highlighting the necessity for a multidisciplinary approach in optimizing patient outcomes. Radiologists and interventional radiologists alike must remain vigilant in the detection and treatment of these rare entities to mitigate the risk of imminent rupture.

## Patient consent

Written, informed consent for the publication and use of images of their case was obtained from the patient.
